# Supplementary material for: 16p11.2 microdeletion imparts transcriptional alterations in human iPSC-derived models of early neural development
Source: eLife. 2020 Nov 10;9:e58178. doi: 10.7554/eLife.58178 (PMC7695459; doi:10.7554/eLife.58178)
Supplement: Transparent reporting form [file elife-58178-transrepform.docx]

***eLife’s* transparent reporting form**

We encourage authors to provide detailed information *within their submission* to facilitate the interpretation and replication of experiments. Authors can upload supporting documentation to indicate the use of appropriate reporting guidelines for health-related research (see [EQUATOR Network](http://www.equator-network.org/%20)), life science research (see the [BioSharing Information Resource](https://biosharing.org/)), or the [ARRIVE guidelines](http://www.plosbiology.org/article/info:doi/10.1371/journal.pbio.1000412) for reporting work involving animal research. Where applicable, authors should refer to any relevant reporting standards documents in this form.

If you have any questions, please consult our Journal Policies and/or contact us: [editorial@elifesciences.org](mailto:editorial@elifesciences.org).

**Sample-size estimation**

- You should state whether an appropriate sample size was computed when the study was being designed
- You should state the statistical method of sample size computation and any required assumptions
- If no explicit power analysis was used, you should describe how you decided what sample (replicate) size (number) to use

Please outline where this information can be found within the submission (e.g., sections or figure legends), or explain why this information doesn’t apply to your submission:

This work focused on characterizing the transcriptomes of human induced pluripotent stem cells (hiPSCs) available within the Simons Foundation Variation in Phenotype collection. In total, 65 clones of hiPSCs were generated from 13 individuals with 16p11.2 copy number variations (CNVs). As described at length in the section, “Reprogramming vector integration induces transcriptional abnormalities”, while initial datasets include all clones, later experiments exclude hiPSC clones with integration of reprogramming factor plasmids (Int+).

There were 4 integration-free (Int-) clones containing 16p11.2 duplications (DUP), 2 clones from one individual and 1 clone each from two individuals. There were 13 hiPSC clones containing 16p11.2 deletions (DEL) from 6 individuals; for 4 of the 6 Int- DEL donors, at least 2 clones were used. There were 7 hiPSC clones containing the wild-type (WT) 16p11.2 locus from 3 individuals; at least 2 clones per individual were used.

Previous studies leveraging hiPSCs to study the etiology of neurodevelopmental disorders have utilized hiPSCs from similar numbers of donors. For example, Marchetto et al. (2010) derived neurons from 2 donors when studying Rett Syndrome, Pasca et al. (2011) derived neurons from 5 donors when studying Timothy Syndrome, Shcheglovitov et al. (2013) derived neurons from 2 donors when studying Phelan McDermid, and Marchetto et al. (2017) derived NPCs and neurons from 8 donors when studying non-syndromic ASD.

**Replicates**

- You should report how often each experiment was performed
- You should include a definition of biological versus technical replication
- The data obtained should be provided and sufficient information should be provided to indicate the number of independent biological and/or technical replicates
- If you encountered any outliers, you should describe how these were handled
- Criteria for exclusion/inclusion of data should be clearly stated
- High-throughput sequence data should be uploaded before submission, with a private link for reviewers provided (these are available from both GEO and ArrayExpress)

Please outline where this information can be found within the submission (e.g., sections or figure legends), or explain why this information doesn’t apply to your submission:

With respect to the RNAseq analyses performed within this study a technical replicate reflects data from the same hiPSC clone independently differentiated and subjected to RNAseq. The RNAseq data contains two levels of biological replicates. The first category reflects the number of individuals (i.e., donors). The second category reflects independent clones generated from the same donor.

RNAseq data from technical replicates was evaluated using the collapseReplicates() function furnished by DESeq2 for the purpose of combining technical replicates in our sample.

Deciding how to handle multiple clones from the same patient is a greater challenge. On one hand, individual clones from a single donor can vary widely in growth rates, differentiation, or other features and these attributes can drift as the cell line is propagated over many cell generations. On the other hand, clones from the same patient will also show correlations that result in principle component clustering of clones from a given donor. This is thought to be due primarily to the unique genetic background of each donor.

Our RNAseq data was examined for both the effect of donor and of clone. This allowed us to agnostically identify the effects of reprogramming plasmid integration and expression on transcription (Figure 4). The data does show correlation between clones from individual donors, but much larger effects were correlated with integration status of the clone. In our differential expression analysis of Int- clones (Figure 6), we apply surrogate variables to perform latent batch corrections across clones to examine the effects of 16p11.2 genotype. In both Figure 4 and Figure 6, the effective number of biological replicates was equal to the number of clones: for the study of integration (n=26 Int-, n=26 Int+), and the study of the effects of 16p11.2 deletion (n=13 DEL, n=7 WT).

The RNAseq data referenced in this publication is deposited in the GEO Repository, accession GSE144736.

With respect to the tri-lineage and neural rosette differentiation studies (Figure 2 and Figure 3), we considered biological replicates to be clones from the same donor, and technical replicates to be distinct differentiations of the same clone. Each lineage differentiation was performed at least 3 times for each clone.

The FACS analysis of hiPSC to NPC differentiation (Supp. Figure 2), a subset of 3 hiPSC clones from 2 unique WT donors and 6 hiPSC clones from 3 unique DEL donors were included. The differentiation, staining, and FACS were performed once.

With respect to quantifying the mitotic index of the differentiated WT and DEL NPCs (Supp. Figure 4), every clone which was differentiated into a neural rosette was stained and at least three images were taken and quantified per clone. Taken together, replicates were as follows:

Int- (WT: 7 clones over 21 images, DEL: 13 clones over 48 images)

Int+ (WT: 1 clone over 3 images, DEL: 21 clones over 87 images)

Finally, information describing the reprogramming and integration status of every clone included in this resource is resented in Supplemental Table 2.

**Statistical reporting**

- Statistical analysis methods should be described and justified
- Raw data should be presented in figures whenever informative to do so (typically when N per group is less than 10)
- For each experiment, you should identify the statistical tests used, exact values of N, definitions of center, methods of multiple test correction, and dispersion and precision measures (e.g., mean, median, SD, SEM, confidence intervals; and, for the major substantive results, a measure of effect size (e.g., Pearson's r, Cohen's d)
- Report exact p-values wherever possible alongside the summary statistics and 95% confidence intervals. These should be reported for all key questions and not only when the p-value is less than 0.05.

Please outline where this information can be found within the submission (e.g., sections or figure legends), or explain why this information doesn’t apply to your submission:

Statistical analysis methods for transcriptomic differential expression analyses are described in the Methods section titled “Assessment of the Impact of Reprogramming Factor Integration”. In short, reported p-values represent FDRs calculated using Benjamini-Hochberg multiple hypothesis testing correction provided by the DESeq2 pipeline, which implements a Wald test for hypothesis testing. The threshold for gene significance was set at 0.05.

Gene Ontology Enrichment in Figure 6 is performed by DAVID. Plotted p-values represent unadjusted p-values calculated by the DAVID implementation of a modified Fisher Exact test. In this figure, color is used to indicate the bar that is significant by Bonferroni-adjusted p-value.

The hypothesis test implemented in Supp. Figure 4, panel A was the Sidak multiple comparisons test performed by GraphPad Prism v7.04. The means for each group are as follows:

Int-, WT: 2.43

Int-, DEL: 2.15

Int+, WT: 0.81

Int+, DEL: 3.18

This information, as well as the p-value and value of n, is found in the figure legend.

All error bars are SEM.

(For large datasets, or papers with a very large number of statistical tests, you may upload a single table file with tests, Ns, etc., with reference to sections in the manuscript.)

**Group allocation**

- Indicate how samples were allocated into experimental groups (in the case of clinical studies, please specify allocation to treatment method); if randomization was used, please also state if restricted randomization was applied
- Indicate if masking was used during group allocation, data collection and/or data analysis

Please outline where this information can be found within the submission (e.g., sections or figure legends), or explain why this information doesn’t apply to your submission:

For the analyses in Figure 4, samples were grouped into Int+ and Int- groups to analyze the effects of integration on the sample. Batch variables, like patient CNV status, were accounted for in model design, as is described in the methods section “Assessment of the Impact of Reprogramming Factor Integration”.

For the analyses in Figure 6, samples were grouped according to patient CNV status, as described in the Methods section “Differential Expression Analysis”. Masking was not used in our experimental analysis as knowledge of patient origin, integration status, and CNV status were necessary for assignation of samples to groups and interpretation of fold changes during analysis.

**Additional data files (“source data”)**

- We encourage you to upload relevant additional data files, such as numerical data that are represented as a graph in a figure, or as a summary table
- Where provided, these should be in the most useful format, and they can be uploaded as “Source data” files linked to a main figure or table
- Include model definition files including the full list of parameters used
- Include code used for data analysis (e.g., R, MatLab)
- Avoid stating that data files are “available upon request”

Please indicate the figures or tables for which source data files have been provided:

We have provided additional metadata for each hiPSC clone in the Supplementary Tables, provided as Excel files.

Raw RNAseq data is available in the GEO repository, accession GSE144736.

All code used for bioinformatic analyses in this paper is available at www.github.com/kmuench/16p_resource (DOI: 10.5281/zenodo.1948176).
